# Supplementary material for: Mechanism of Caspase-1 Inhibition by Four Anti-inflammatory Drugs Used in COVID-19 Treatment
Source: Int J Mol Sci. 2022 Feb 6;23(3):1849. doi: 10.3390/ijms23031849 (PMC8837144; doi:10.3390/ijms23031849)
Supplement: Supplementary file 1 [file ijms-23-01849-s001.zip › ijms-1571540-Supplementary.pdf]

Supplementary Figures

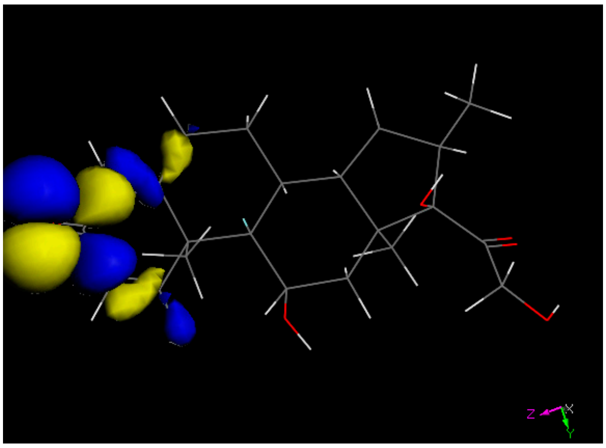

Figure S1. HOMO electron density of dexamethasone is concentrated in the methide-quinone moiety, left.

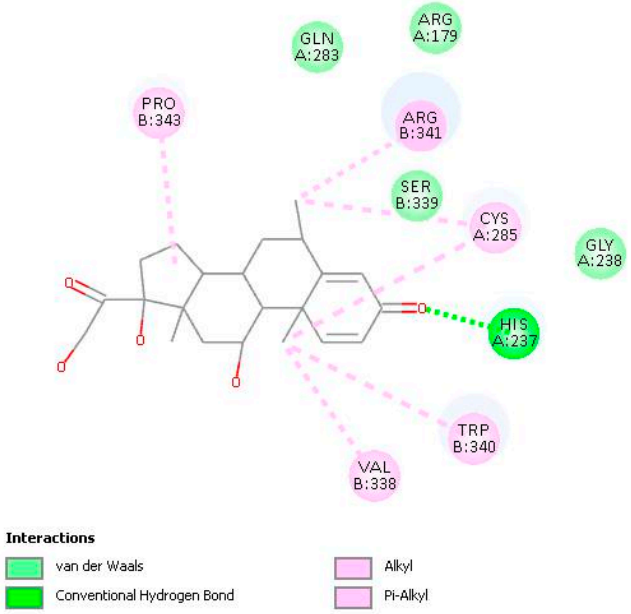

Figure S2. 2D display of amino acid interactions of docked methylprednisolone pose 8.

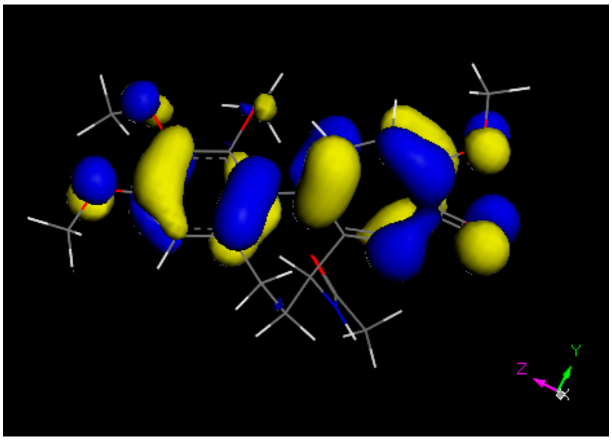

Figure S3. HOMO of colchicine. The tropolone carbonyl is located to the right. It is seen that the orbital expansion of 6- and 7-membered rings are similar. The acetamide carbonyl, bottom, does not appear involved in potential interaction, when compared with the tropolone carbonyl, right.

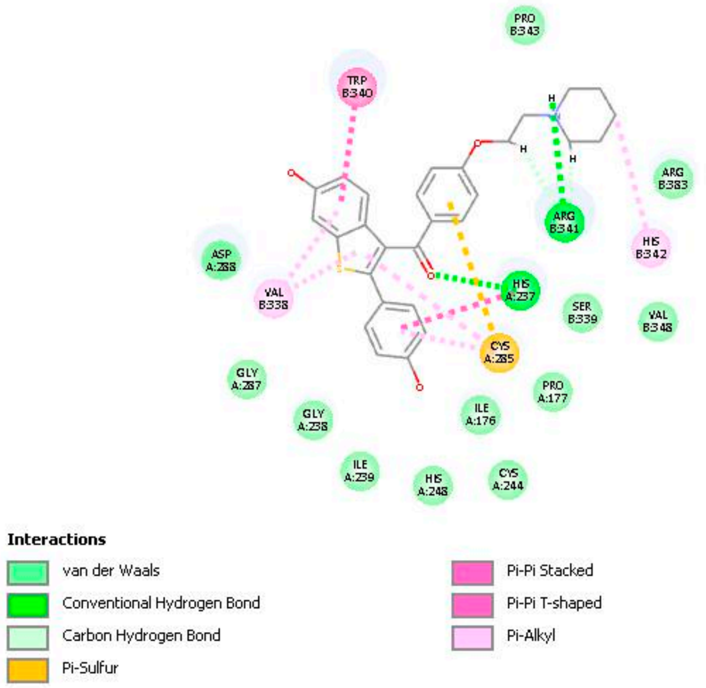

Figure S4. 2D display of amino acid interactions of docked raloxifene pose 2. Interestingly, the approach of S(Cys285) to raloxifene C(carbonyl) seems facilitated by an aromatic ring interaction adjacent to carbonyl. Additional stabilization is provided by a π-π T-shaped Trp-B-340 to the aromatic ring located top left.

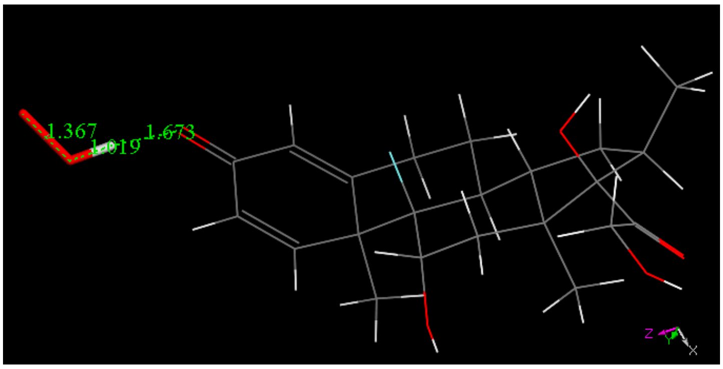

Figure S5. Protonated dexamethasone—O_2Radical σ approach. A classical scavenging of superoxide, which captures an H from a ring hydroxyl substituent. This picture helps to explain the initial non-flat part of the RRDE experiment, where humidity in DMSO may allow formation of a protonated dexamethasone carbonyl $[C=OH]^+$. Thus, superoxide captures this proton and consequently less superoxide is detected at the ring electrode. Once the $[C=OH]^+$ moiety is exhausted, superoxide remains stable (not scavenged), with slope zero in the Efficiency graph shown below.

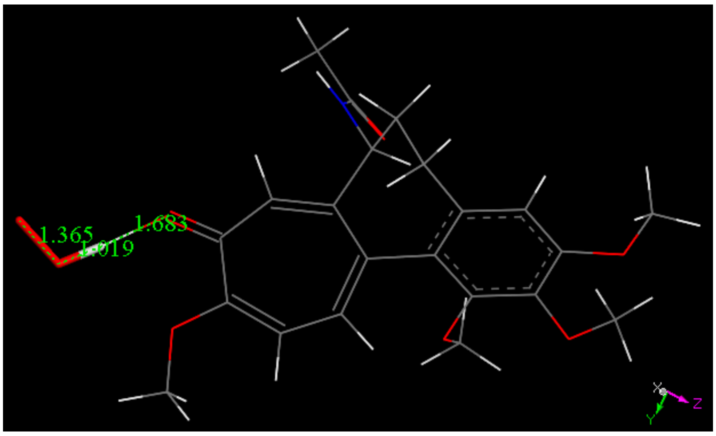

Figure S6. Protonated colchicine—superoxide radical, σ approach. A classical scavenging of superoxide, which captures an H from a ring hydroxyl substituent. This picture suggests a decreasing behavior of the efficiency in the RRDE experiment, i.e. decrease of superoxide concentration. We conclude that, based on DFT results, the colchicine tropolone ring is a better scavenger of superoxide than the methide-quinone ring of dexamethasone, which suggests that tropolone ring can, probably, establish a good covalent bond with S(Cys285) thiolate.
